# The Traditional Chinese Medicine DangguiBuxue Tang Sensitizes Colorectal Cancer Cells to Chemoradiotherapy

**DOI:** 10.3390/molecules21121677

**Published:** 2016-12-06

**Authors:** Shun-Ting Chen, Tzung-Yan Lee, Tung-Hu Tsai, Yin-Cheng Lin, Chin-Ping Lin, Hui-Ru Shieh, Ming-Ling Hsu, Chih-Wen Chi, Ming-Cheng Lee, Hen-Hong Chang, Yu-Jen Chen

**Affiliations:** 1 Department of Chinese Medicine, Buddhist Tzu Chi General Hospital, Taipei Branch, New Taipei City 23142, Taiwan; cst.tp.tw@gmail.com; 2 Graduate Institute of Traditional Chinese Medicine, School of Chinese Medicine, College of Medicine, Chang Gung University, Taoyuan 33302, Taiwan; joejoylee@gmail.com; 3 Graduate Institute of Clinical Medical Science, College of Medicine, Chang Gung University, Taoyuan 33302, Taiwan; 4 Institute of Traditional Medicine, School of Medicine, National Yang-Ming University, Taipei 11221, Taiwan; thtsai@ym.edu.tw; 5 Department of Medical Research, Mackay Memorial Hospital, Taipei 25160, Taiwan; eric7772330@gmail.com (Y.-C.L.); cplin66@gmail.com (C.-P.L.); ru123@ms1.mmh.org.tw (H.-R.S.); sabinehsu@gmail.com (M.-L.H.); cwchid48906003@gmail.com (C.-W.C.); 6 Department of Research, Buddhist Tzu Chi General Hospital, Taipei Branch, New Taipei City 23141, Taiwan; sardorna@yahoo.com.tw; 7 School of Post-Baccalaureate Chinese Medicine, and Research Center for Chinese Medicine and Acupuncture, China Medical University, Taichung 40402, Taiwan; 8 Department of Chinese Medicine, China Medical University Hospital, Taichung 40402, Taiwan; 9 Department of Radiation Oncology, Mackay Memorial Hospital, Taipei 25160, Taiwan

**Keywords:** DangguiBuxue Tang, colorectal cancer, chemotherapy, radiotherapy

## Abstract

Chemotherapy is an important treatment modality for colon cancer, and concurrent chemoradiation therapy (CCRT) is the preferred treatment route for patients with stage II and III rectal cancer. We examined whether DangguiBuxue Tang (DBT), a traditional Chinese herbal extract, sensitizes colorectal cancer cells to anticancer treatments. The polysaccharide-depleted fraction of DBT (DBT-PD) contains greater amounts of astragaloside IV (312.626 µg/g) and ferulic acid (1.404 µg/g) than does the original formula. Treatment of the murine colon carcinoma cell line (CT26) with DBT-PD inhibits growth, whereas treatment with comparable amounts of purified astragaloside IV and ferulic acid showed no significant effect. Concurrent treatment with DBT-PD increases the growth inhibitory effect of 5-fluorouracil up to 4.39-fold. DBT-PD enhances the effect of radiation therapy (RT) with a sensitizer enhancement ratio (SER) of up to 1.3. It also increases the therapeutic effect of CCRT on CT26 cells. Cells treated with DBP-PD showed ultrastructural changes characteristic of autophagy, including multiple cytoplasmic vacuoles with double-layered membranes, vacuoles containing remnants of degraded organelles, marked swelling and vacuolization of mitochondria, and autolysosome-like vacuoles. We conclude that DBT-PD induces autophagy-associated cell death in CT26 cells, and may have potential as a chemotherapy or radiotherapy sensitizer in colorectal cancer treatment.

## 1. Introduction

Colorectal cancer (CRC) is globally the second and third most commonly diagnosed cancer in women and men, respectively [1]. Approximately 70% of cases arise in the colon [2] and 80% of cancers are localized on the colon wall and/or regional nodes, and surgical resection is the only curative modality for localized colon cancer. Downstaging of rectal cancer before surgery by neoadjuvant chemoradiotherapy remains the standard procedure for improving survival of patients with locally advanced lesions. Radiotherapy (RT) is used in rectal cancer for local control and as a neoadjuvant treatment, but its success rate in achieving pathological complete response (pCR) still needs improvement. Coupling RT with chemotherapy can help to control locoregional recurrence and distant metastases, but the overall success rate remains unsatisfactory. The 5-year survival rates for stage IIIc and stage IV CRC are only 28.0% and 5.7%, respectively [3]. These data explain the pressing need for development of RT and chemotherapy enhancers. Some chemotherapeutics, such as 5-fluorouracil, are currently used as RT sensitizers, but these are associated with significant negative side effects, including mucositis, emesis, diarrhea, febrile neutropenia, and fatigue [4,5]. New RT enhancers with reduced toxicity are necessary. 

Development from traditional Chinese medicine (TCMs) of enhancers such as PHY-906 [6,7] or natural products is a growing field. DangguiBuxue Tang (DBT) is a Chinese herbal decoction traditionally used to treat menstrual anemia. It contains *Astragali Radix* (AR) and *Angelicae Sinensis* Radix (ASR) in a 5:1 ratio, as described in 1247 A.D by Dr. Dong-Yuan Li [8]. Practitioners of traditional Chinese medicine use DBT to reinforce qi and nourish blood. Pharmacological results revealed that DBT may enhance hematopoietic function by up-regulating erythropoietin (EPO) production and bone marrow cell activity [9,10,11]. This enhancement of hematopoietic function is one reason why traditional Chinese medicine practitioners prescribe DBT for cancer patients after surgery or during chemo-radiotherapy. Estrogenic effects of DBT through phosphorylation of estrogen receptor (ER-α) and extracellular signal-regulated kinase 1/2 (Erk1/2) [12] have been reported, suggesting clinical usefulness in post-menopausal women to relieve vasomotor symptoms [13]. Promotion of osteoproliferation and osteoblast differentiation is another important activity of DBT useful in osteoporosis patients [14,15]. Thus far, 54 chemical components have been identified in DBT decoction. Despite this knowledge, our understanding of DBT’s mechanism(s) of action is still very limited [16].

In previous studies, DBT enhanced the growth of osteosarcoma MG-63 [14] and breast cancer MCF-7 cells [12] in vitro. Chiu et al. also demonstrated that DBT protected H9c2 cells against oxidative injury by increasing levels of reduced cellular glutathione [17]. Anti-oxidants such as glutathione could compromise the effect of RT. These studies raise concerns regarding clinical application of DBT in cancer patients. In the present study, we examined the effect of DBT on tumor cell viability, radiosensitivity, and sensitivity to chemotherapy to clarify the potential of DBT as an anti-cancer treatment.

## 2. Results

### 2.1. Qualitative and Quantitative Analysis of Ferulic Acid and Astragaloside IV in DBT

**Figure 1:**
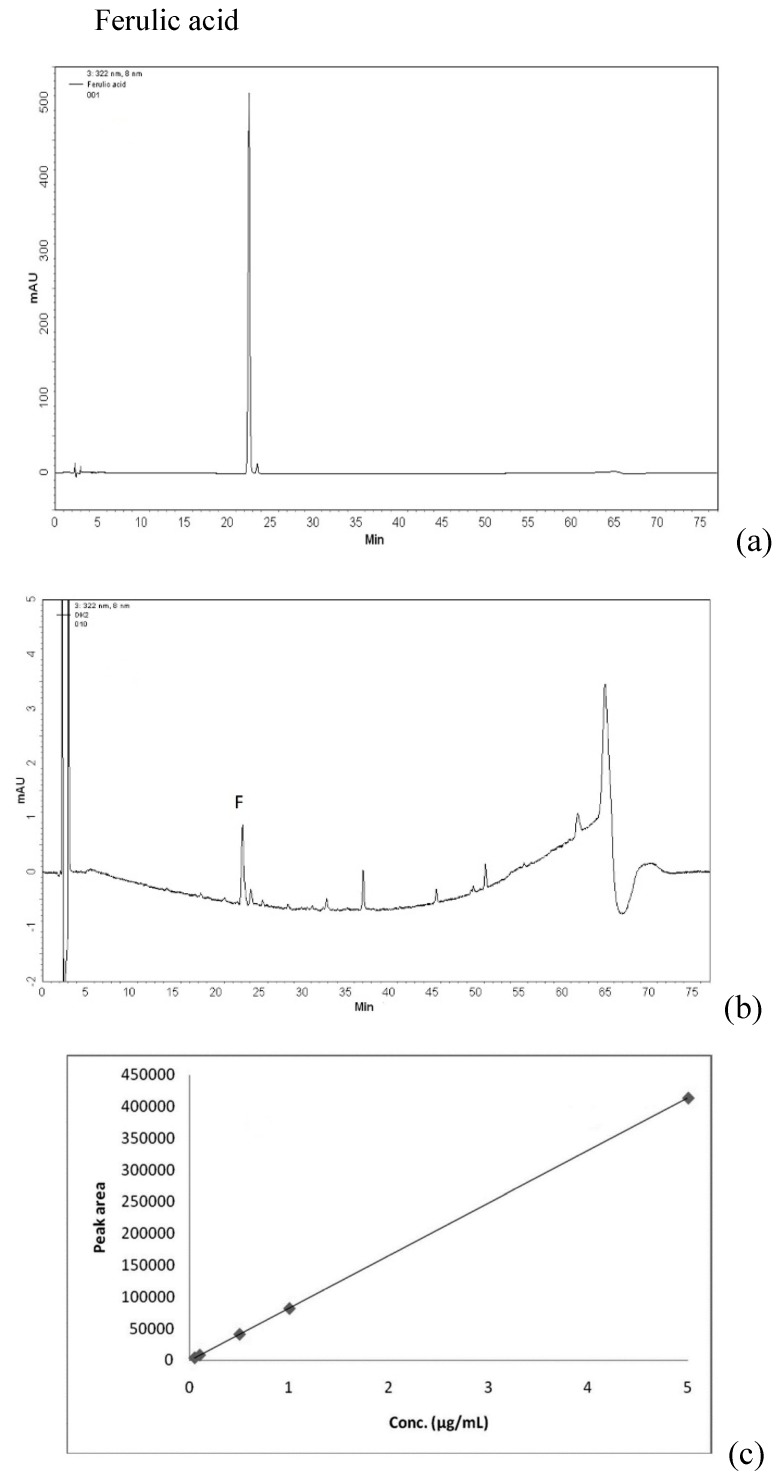

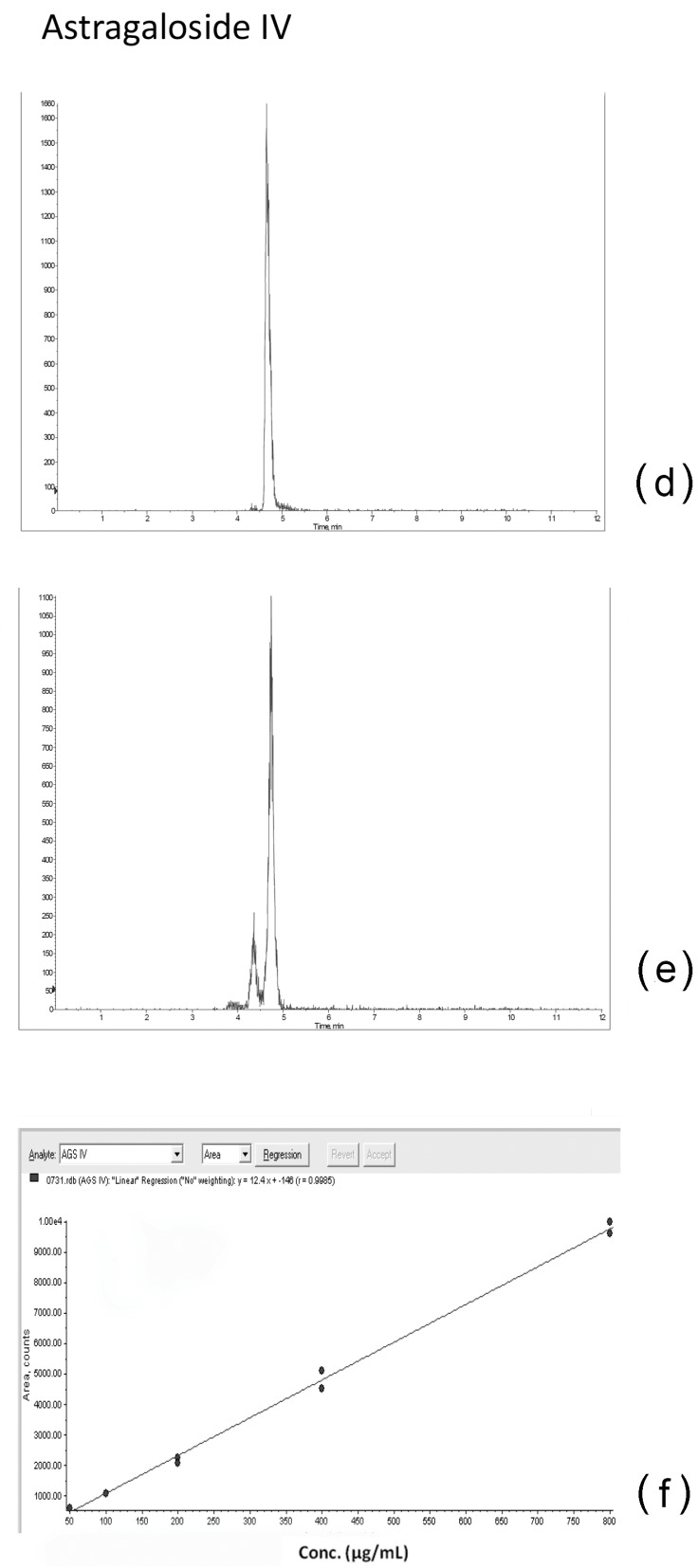
Qualitative and quantitative analysis of ferulic acid and astragaloside IV by HPLC. (**a**) A chromatogram of the ferulic acid standard. Its retention time is 22.7 min; (**b**) A chromatogram of DBT-PD. The retention time of its ferulic acid is 23.1 min; (**c**) Calibration curve (0.05–5 µg/mL) of ferulic acid; (**d**) Multiple reaction monitoring (MRM) mass spectrometric chromatogram of the astragaloside IV standard. Its retention time is 4.75 min; (**e**) MRM chromatogram of astragaloside IV in DBT-PD. Its retention time is 4.76 min; (**f**) Calibration curve (0.5–8 µg/mL) of astragaloside IV.

DBT, a 5:1 weight:weight mixture of AR and ASR, was prepared using extraction conditions optimized in a previous study [16]. To chemically standardize the herbal extract and ensure batch-to-batch consistency, we generated HPLC fingerprints based on astragaloside IV to gauge relative amounts of AR, and ferulic acid to gauge relative amounts of ASR (Figure 1). Amounts of astragaloside IV in dried powders of DBT, polysaccharide-enriched fraction (DBT-PE) and a polysaccharide-depleted (DBT-PD) were 48.46 µg/g, 11.37 µg/g, and 312.63 µg/g, respectively. Amounts of ferulic acid were 0.29 µg/g, 0.43 µg/g, and 1.40 µg/g, respectively (Table 1).

*Astragali Radix* (AR) and *Angelicae Sinensis* Radix (ASR) were extracted in boiling water, and DBT was separated into a polysaccharide-enriched (DBT-PE) fraction and a polysaccharide-depleted (DBT-PD) fraction by precipitating with 50% ethanol. Ferulic acid and astragaloside IV are the index components of AR and ASR that were detected by HPLC-UV and HPLC-MS. DBT-PD contained the highest amounts of astragaloside IV (312.626 µg/g) and ferulic acid (1.404 µg/g).

### 2.2. Osmolality of Culture Medium Containing DBT-PD

As shown in Table 2, osmolality of culture medium without DBT-PD was 291.0 ± 5.2 mOsm/kg. Adding ethanol-dissolved DBT-PD cell culture media at a final concentration of less than 15 mg/mL elevated the osmolality to 387.3 ± 14.4 mOsm/kg. Increasing DBT-PD concentration to 30 mg/mL further elevated osmolality to 507.7 ± 18.8 mOsm/kg. To ensure that any inhibition of cell viability was not due to osmolality, the concentration of DBT-PD used in the present study was ≤ 10 mg/mL. The osmolality at this concentration was comparable to that of the media containing ethanol alone (360.0 ± 21.7 versus 375.3 ± 34.1 mOsm/kg, *p* > 0.05).

### 2.3. Inhibition of Viability of CT26 and HT-29 Cells by DBT-PD

**Figure 2:**
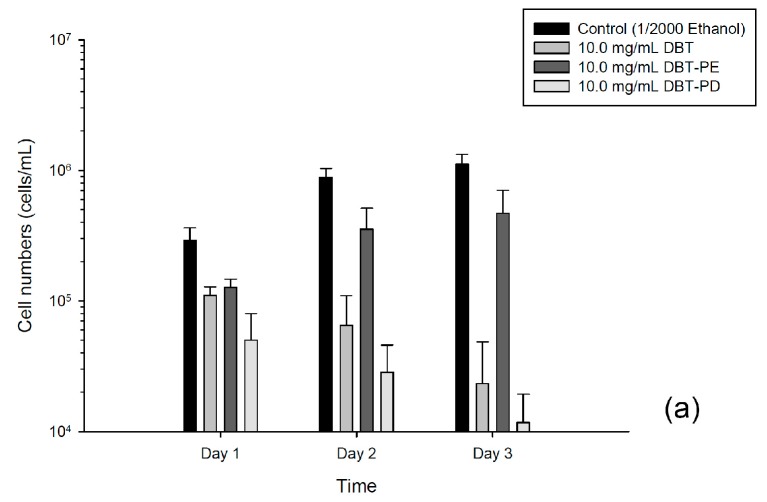

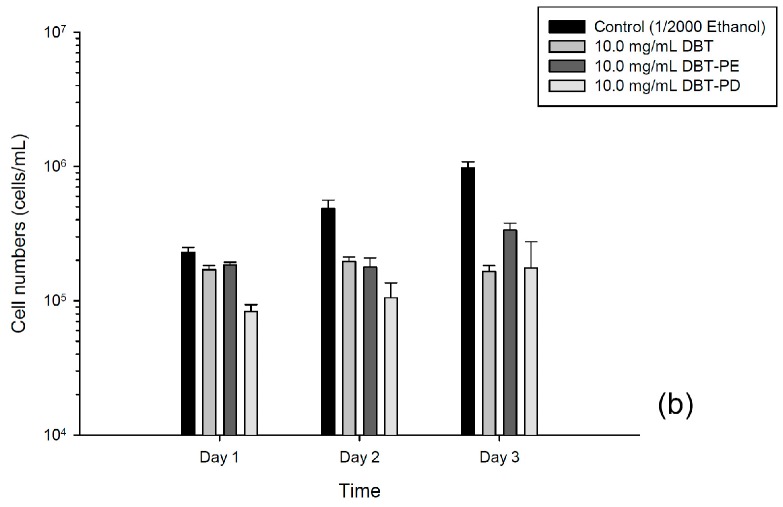

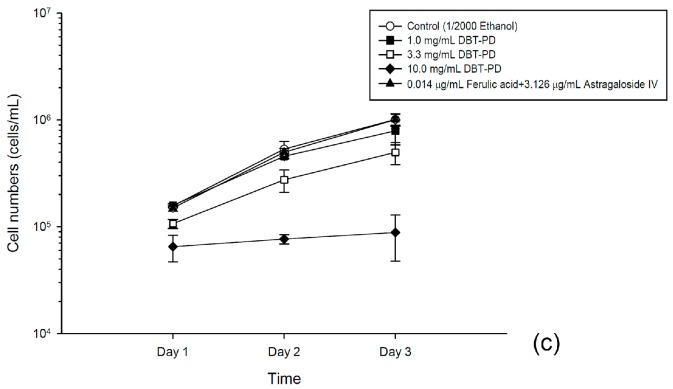

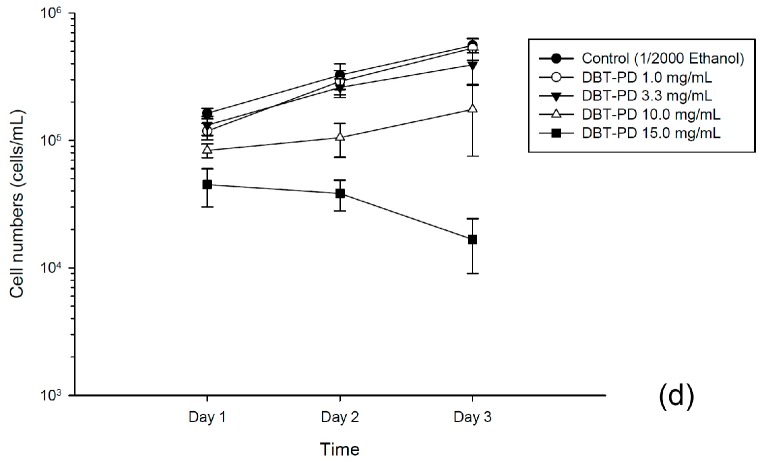

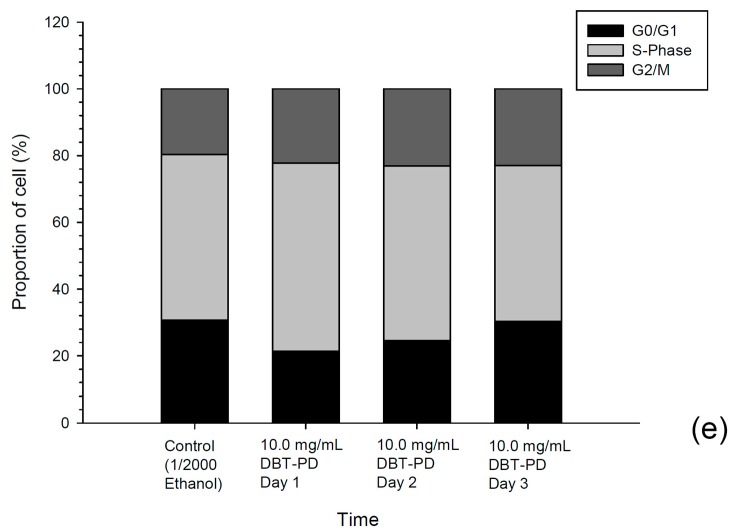

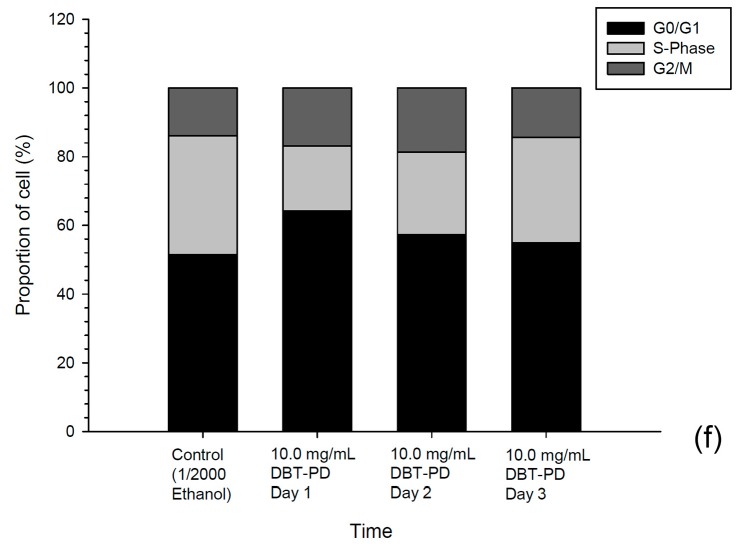
Viability and cell cycle analysis of CT26 and HT-29 cells after treatment with DBT. (**a**,**b**) Cells were cultured with 10.0 mg/mL DBT, DBT-PE, or DBT-PD for 24, 48 and 72 h. Cell viability was measured by Trypan blue dye exclusion; (**c**,**d**) Cells were cultured without or with DBT-PD (0–15.0 mg/mL) for 24, 48 and 72 h. Viable cells were counted by trypan blue exclusion assay. Data from at least three separate experiments are expressed as mean ± standard deviation; (**e**,**f**) Cell cycle analysis in CT26 cells treated with 10 mg/mL DBT-PD.

Treatment with 10 mg/mL of DBT or DBT-PD extracts, but not DBT-PE, inhibited the viability of CT26 cells in a time-dependent manner (Figure 2a). DBT-PD showed the greatest growth inhibitory activity. It inhibited the growth of CT26 cells in a dose- and time-dependent manner with an estimated IC_50_ of 2.98 mg/mL after 24 h and 2.05 mg/mL after 72 h (Figure 2c). Intriguingly, purified astragaloside IV (3.126 µg/mL) and ferulic acid (0.014 µg/mL), in amounts equivalent to that contained in 10 mg/mL DBT-PD, had no significant growth inhibitory effect (Figure 2c). This result implies that these two index components are not the primary active ingredients of DBT-PD that contribute to its inhibitory effects on growth of CT26 cells. Next, we examined the effects of DBT, DBT-PD, and DBT-PE on human colorectal adenocarcinoma HT-29 cells. The growth inhibitory effect of these three extractions was shown at 48 and 72 h; however, it was to a lesser extent than that observed in CT26 cells (Figure 2b). DBT-PD inhibited HT-29 cell growth in a time- and dose-dependent manner (Figure 2d). The IC_50_ of DBT-PD on HT-29 cells at 24 and 72 h were 10.1 mg/mL and 7.3 mg/mL, respectively. This implies that DBT-PD also inhibited cell growth in human colorectal adenocarcinoma.

### 2.4. Cell Cycle Assay

DNA histograms of treated and control cells showed that significant changes in the distribution throughout the cell cycle after DBT-PD treatment was observed in neither CT26 cells nor HT-29 cells (Figure 2e,f).

### 2.5. Caspase Activities

To understand the role of apoptosis involved in DBT-PD-mediated cell death, activities of various caspases, including initiator and executioner caspases, were measured. Results showed no significant difference between control cells and DBT-PD-treated cells (Table 3). This indicates that apoptosis may not be involved in DBT-PD-induced cell death.

### 2.6. Cell Morphology

**Figure 3:**
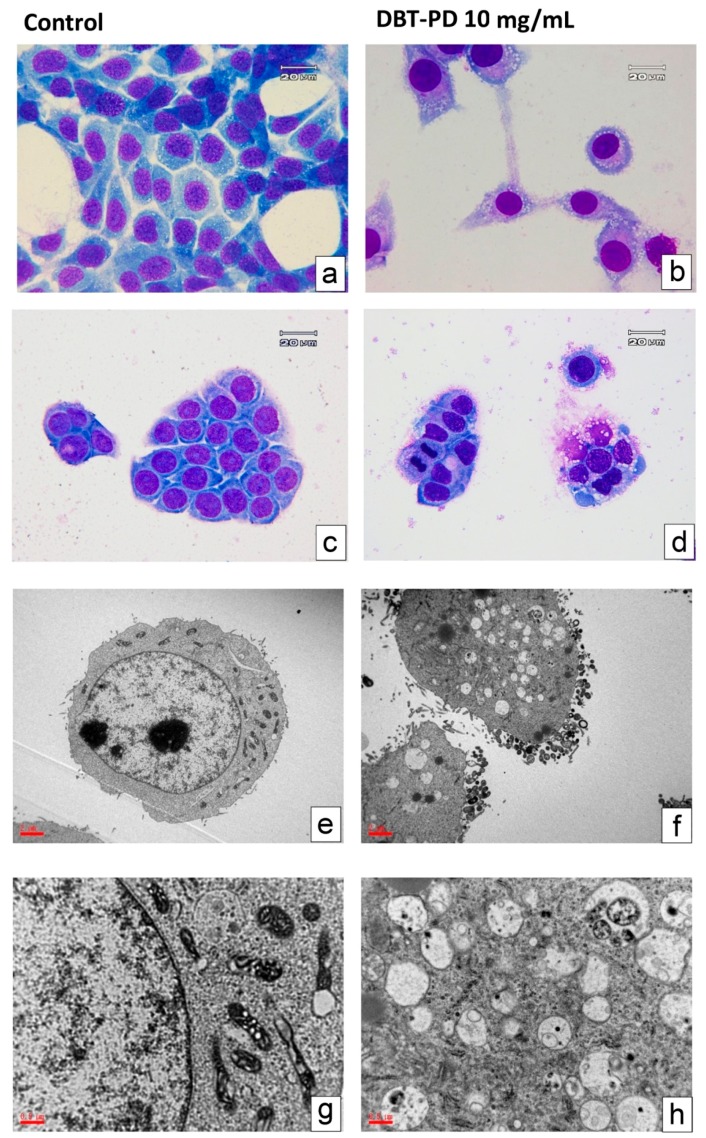
Morphology changes in CT26 cells. Left panels: CT26 cells without treatment Right panels: CT26 cells treated with 10mg/mL DBT-PD for 72h. (**a**,**b**) CT26 cells; (**c**,**d**) HT-29 cells were stained with Liu’s stain and photographed under a light microscope; (**e**–**h**) TEM microphotographs showing the ultrastructure of CT26 cells. Magnification 10,000- and 20,000-fold.

Light microscopy analysis of CT26 cells treated with DBP-PD (10 mg/mL for 72 h) showed the presence of abundant cytoplasmic vacuoles relative to control cells (Figure 3a,b). Similar results were obtained in human colorectal adenocarcinoma HT-29 cells treated with DBP-PD compared with control cells (Figure 3c,d). Transmission electron microscopy revealed that treatment of CT26 cells with DBT-PD caused changes characteristic of autophagy, including appearance of multiple cytoplasmic vacuoles with double-layered membranes (Figure 3f,h).

Vacuoles resembling autophagosomes and containing remnants of degraded organelles were noted. Marked swelling of mitochondria with vacuolization was observed in DBT-PD-treated cells, and autolysosomes were observed.

### 2.7. Enhancement of 5-Fluorouracil (5-FU) Effect by DBT-PD

Concurrent treatment of CT26 cells with DBT-PD and low concentrations of 5-fluorouracil (5-FU) markedly inhibited cell growth compared to that with DBT-PD or 5-FU alone (Table 4). It is noteworthy that DBT-PD markedly enhanced the growth inhibitory effect of 0.1 and 0.5 µM 5-FU than that of 1.0 µM 5-FU alone. The combination indices (CI) were calculated using CompuSyn software for both concurrent treatment with DBT-PD and 5-FU, which are shown in the Table 4. For the HT-29 cells, concurrent treatment of cells with DBT-PD and 5-FU did not show additive effect for growth inhibition compared to that with DBT-PD or 5-FU alone on day 1 and day 2 (Table 5). At 72 h, 1.0 mg/mL DBT-PD markedly enhanced the growth inhibition of 0.1 and 0.5 µM 5-FU than that of 1.0 µM 5-FU alone.

### 2.8. The Sensitizing Effect of DBT-PD on RT and CCRT

**Figure 4:**
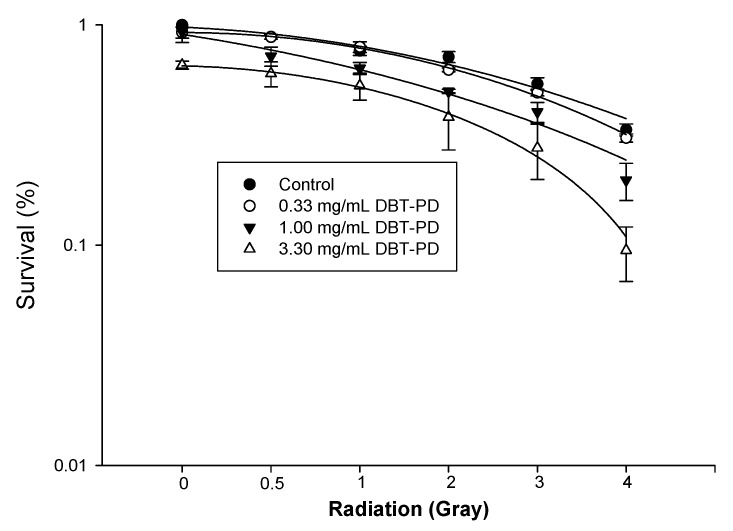
Radiation survival curve of CT26 cells. CT26 cancer cells were treated with 0.33, 1.00, and 3.30 mg/mL DBT-PD for 24 h before irradiation. Colony formation assay was performed for estimation of radiation survival. Data from 3 experiments are presented as mean ± standard deviation.

**Figure 5:**
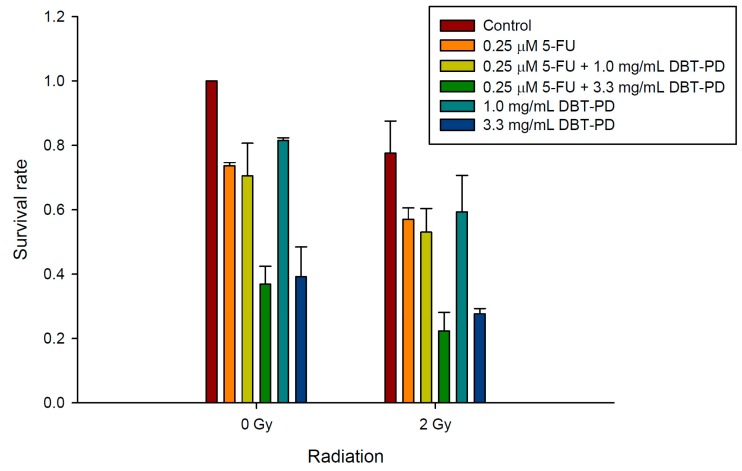
Effect of DBT-PD on survival of CT26 cells subjected to CCRT. CT26 cancer cells were treated with 0.25 μM 5-FU and 1.0 or 3.3 mg/mL DBT-PD for 24 h before 2-Gy irradiation. A colony formation assay was performed to estimate survival post CCRT. Data from 3 experiments are presented as mean ± standard deviation.

Irradiation of untreated CT26 cells at a dose of 0 to 4 Gy killed 66.6% of cells. Pretreatment with 0.33, 1.00, and 3.30 mg/mL DBT-PD markedly decreased the survival of irradiated tumor cells (Figure 4). The estimated sensitizer enhancement ratios (SER) were 1.1 and 1.3 for doses of 1.00 and 3.30 mg/mL, respectively. In our preliminary in vivo experiment, both control and irradiation (RT) groups did not show tumor growth inhibition. However, in vivo, the oral administration of DBT-PD markedly inhibited the tumor growth. Unfortunately, DBT-PD did not enhance the RT effect on tumor growth (Supplementary Materials Figure S1). Next, we evaluated the sensitizing effect of DBT-PD on CCRT in CT26 cells, relevant to clinical treatment for rectal cancer. Colony formation assays indicated that DBT-PD enhanced the effect of CCRT (0.25 μM 5-FU and 2-Gy RT) (Figure 5).

## 3. Discussion

DBT, an ancient Chinese herbal formula for menstrual anemia, was shown to inhibit the growth of and enhance efficacy of RT, 5-FU, and CCRT in CT26 cells. In clinical practice, TCM physicians frequently use DBT to manage general deficiencies of qi and blood. For patients receiving anti-cancer treatments, DBT is prescribed to reduce fatigue and promote recovery from adverse effects related to anti-cancer treatments. However, ambiguities exist that raise concerns about interactions among DBT, cancer cells, and anti-cancer treatments. While the beneficial effects of DBT have been reported in some cancer cells [12,14,17], TCM physicians have been considering the possible benefits of incorporating this ancient formula into modern anti-cancer treatment modalities. In this study, we used colorectal cancer CT26 cells as an experimental model to examine the effects of DBT. Our results show that DBT, especially the polysaccharide-depleted fraction, may play a beneficial role in anti-cancer treatments.

HPLC data showed that DBT-PD contains greater amounts of index components (astragaloside IV and ferulic acid) than do DBT and DBT-PE. In apparent correspondence with this observation, DBT-PD inhibited growth with greater potency than did DBT and DBT-PE. However, purified astragaloside IV and ferulic acid at amounts equivalent to those found in DBT-PD did not have a similar effect on CT26 cells. Gao et al. demonstrated that the polysaccharide-enriched fraction of DBT showed marked responses in cultured T-lymphocytes, suggesting an important role for DBT polysaccharides in triggering such immune responses [18]. Taken together, these observations suggest the presence of CT26 cell targeting components other than polysaccharides in DBT.

On the basis of morphology changes in CT26 cells treated with DBT-PD, a form of autophagic cell death was induced. This is a novel finding regarding the activity of DBT in cancer cells. Autophagy (or type 2 cell death) is a highly regulated process that can be involved in turnover of long-lived proteins and whole organelles, or can target specific organelles (for example, mitochondria in mitophagy and the endoplasmic reticulum (ER) in reticulophagy) to eliminate superfluity or damaged organelles [19,20]. During autophagy, parts of the cytoplasm and intracellular organelles are engulfed within characteristic double- or multi-membraned autophagic vacuoles (named autophagosomes) and delivered to lysosomes for bulk degradation. Autophagy is also a metabolic response to starvation, and can be triggered by decreased extracellular nutrients or decreased intracellular metabolite concentrations that result from the loss of growth-factor signaling (which often governs the uptake of nutrients). By catabolism of macromolecules, autophagy generates metabolic substrates that help meet the bio-energetic needs of cells and allow for adaptive protein synthesis [21]. In this study, after treatment with 10 mg/mL DBT-PD, abundant cytoplasmic vacuoles were observed under the light microscope, which resemble autophagosomes or autolysosomes. Multiple cytoplasmic vacuoles with double layer membranes and vacuoles containing remnants of degraded organelles including swollen mitochondria could be identified as autophagosomes by using TEM. Autolysosomes could also be detected using TEM. The detailed pathway of autophagic cell death and related molecular events still needs be investigated.

The sensitization effect of DBT-PD for RT and chemotherapy is unique. The mechanisms remain to be determined. Cell cycle analysis showed no G2/M arrest. Although a mild increase in sub G1 cells was noted, no apoptotic bodies or other apoptosis-induced changes in morphology could be found. Radiation-induced autophagy is believed to represent a radioprotective mechanism in cancer cells. Because of the ability of autophagy to remove damaged proteins or organelles, it may paradoxically serve as a mechanism for promoting survival of irradiated cells. Thus, inhibition of autophagy should support radiation treatment and increase its efficacy [22]. However, there are evidences that radiation alone or in combination with various chemical agents can induce autophagy that increases cell death, especially within transformed apoptosis-resistant cells [23]. In this study, DBT-PD seems to act as a radiosensitizer, possibly by inducing autophagy. A similar effect is seen when DBT is used in combination with chemotherapy drugs. DBT-PD has the potential to enhance the effect of RT or chemotherapy or both.

In this study, we demonstrate the novel anti-neoplastic effect of DBT alone and combined with RT or chemotherapy. Autophagy seems to be the main mechanism of DBT-mediated cytotoxicity, but many questions remain to be clarified. First, pathways involving beclin-1, mTOR, Atg proteins among others [24] that play a role in autophagy need to be investigated. Second, whether the phenomena observed in this in vitro study will translate into in vivo models remains unknown. Further mechanistic and in vivo studies are warranted.

## 4. Materials and Methods 

### 4.1. Plant Materials and Preparation of DBT

The traditional Chinese medicines *Astragali Radix* (AR) and *Angelicae Sinensis* Radix (ASR), were kindly provided by the Chinese medicine manufacturer Sun Ten Pharmaceutical Co. Ltd. in New Taipei City, Taiwan. Authentication of AR and ASR were performed according to Doc No. BR3-TE01 Authentication SOP by Non-profit organization Brion Research Institute of Taiwan, New Taipei City, Taiwan. Before extraction, *Angelicae Sinensis* Radix was immersed in rice wine (alcohol 75% *v*/*v*) overnight, and the pedicles were removed in accordance with the traditional method of preparing the Chinese medicine. The preparation methods of DBT followed the ancient formula designed to optimize extraction [16]. DBT, consisting of AR and ASR in the ratio of 5:1, was boiled in water for 1 h, and separated into a polysaccharide-enriched fraction (DBT-PE) and a polysaccharide-depleted (DBT-PD) fraction by precipitation in 50% ethanol. Both fractions were concentrated by lyophilization, but only DBT-PD, the fraction with greater bioactivity, was further investigated in these experiments. 

### 4.2. Chemical Standardization of DBT

The amounts and purity of ferulic acid and astragaloside IV in DBT were determined separately using HPLC-UV and HPLC-MS systems by comparison with standards for ferulic acid (ChromaDex, Irvine, CA, USA) and astragaloside IV (Tauto Biotech, Shanghai, China). In the HPLC-UV system, an RP-C18 column (250 mm × 4 mm, 5 µm particle size, Purospher® STAR, Merck, Darmstadt, Germany) was used, with a mobile phase consisting of 90% methanol and 4% acetic acid in water and a flow rate of 1.0 mL/min. Absorbance was measured at 322 nm. In the HPLC-MS system, aC18 column (50 mm × 2 mm, 5 µm particle size, Phenomenex, Torrance, CA, USA) was used, with a mobile phase consisting of 50% methanol (A) and a stationary phase (B) consisting of 5% ammonium acetate in water, at a flow rate of 250 µL/min. The metabolite was eluted with a gradient constructed as follows: 0–0.5 min, 70% A and 30% B; 2–8 min 5% A and 95% B, then A and B are returned to initial conditions and the metabolite is detected by mass spectrometry. An Applied Biosystems 3200 Q TRAP LC/MS/MS system (Applied Biosystems, Waltham, MA, USA) equipped with electrospray was used. The conditions of mass spectrometric data acquisition in the positive ion model were ion spray voltage 5.5 KV; gas temperature 400 °C; *m*/*z*: 785.1; fragment ions *m*/*z*: 143.2; declustering potential voltage 50 V; collision energy 25 V; and collision cell exit potential 2 V.

### 4.3. CT26 and HT-29 Cell Culture

The mouse colorectal adenocarcinoma cell line CT26 and human colorectal adenocarcinoma cell line HT-29 were purchased from the American Type Culture Collection (Manassas, VA, USA) and maintained according to the culturing methods recommended by ATCC. CT26 cells were cultured in RPMI-1640 medium (Gibco, Grand Island, New York, NY, USA) supplemented with 10% heat-inactivated fetal calf serum (FCS; Hyclone, Logan, UT, USA) in a humidified incubator containing 5% CO_2_ at 37 °C. For HT-29 cells, cells were cultured in McCoy’s 5A medium supplemented with 10% heat-inactivated FCS. Cells were subcultured every 2–3 days with Trypsin–EDTA–glucose solution (0.25% trypsin, 0.1% EDTA, and 0.05% glucose in Hanks’ balanced salt solution), and maintained in exponential growth.

### 4.4. Determination of Osmolality in Culture Medium 

Osmolality of culture medium with or without different concentrations of DBT-PD was measured using a micro-osmometer (Advanced Instruments, Inc., Norwood, MA, USA). The methods of measuring medium osmolality were based on freezing point depression. For accuracy and precision, commercial calibration standards were prepared for each measurement.

### 4.5. Cell Viability Assays

CT26 cells and HT-29 cells (10^5^ cells/mL) were cultured in 3.5 cm dishes for 24 h, and then treated with concentrations of DBT-PD ranging from 0 to 30 mg/mL for 24, 48, and 72 h. After incubation ± DBT, numbers of viable cells were counted using a trypan blue dye exclusion test. To assess synergistic effects of DBT-PD with chemotherapy, 5-fluorouracil (5-FU) at concentrations ranging from 0 to 1 µM, and 0 to 3.3 mg/mL DBT were added simultaneously into CT26 or HT-29 cells cultures and cell viability was assayed at 24, 48, and 72 h.

### 4.6. Cell Cycle Analysis by Flow Cytometry

Cellular DNA content was measured using propidium iodide (PI) staining and flow cytometry. After treatment with 10 mg/mL DBT-PD for 24, 48, and 72 h, cells were harvested, washed with phosphate buffered saline (PBS), and fixed with ice-cold 70% ethanol for at least 30 min. Cells were incubated with 0.1% Triton X-100, 0.5 mg/mL RNase A (Sigma-Aldrich Corp., St. Louis, MO, USA), and 10 mg/mL PI for 30 min at 37 °C in the dark. The DNA histogram for cell cycle distribution was analyzed by a FACSCalibur flow cytometer (Becton Dickinson, Lincoln Park, NJ, USA). Data from 10,000 cells were collected and the percentage found in each phase of the cell cycle was determined using CellQuest cell cycle analysis software (version 2.01.2 Becton Dickinson, Franklin Lakes, NY, USA).

### 4.7. Measurement of Caspase Activities

Caspase activities were determined using the Caspase Fluorometric Substrate Set II Plus assay kit and the detailed experiments were performed according to the manufacturer’s protocol (MBL, Nagoya, Aichi, Japan). Briefly, after treatment, cells were harvested and lysed with cell lysis buffer. Supernatants were collected and loaded with equal amounts of protein samples to react with the AFC-conjugated substrates. The caspase activities were measured using a fluorometer equipped with a 400-nm excitation and 505-nm emission filter.

### 4.8. Morphological Observation by Light and Transmission Electron Microscopy

Murine CT26 and human HT-29 cells treated with DBT-PD for 3 days were collected and stained with Liu’s stain solution. Cell morphology was observed under an Olympus light microscope at a magnification of 1000-foldand photographed with a charged-couple device camera. For observation under a transmission electron microscope, CT26 cells were washed and fixed with 2.5% glutaraldehyde in 0.1 M cacodylate buffer for 2 h. After washing with PBS, cells were post-fixed with 1% osmium tetroxide for 1 h and dehydrated with alcohol and acetone. Samples were embedded in Spurr’s resin, semithin sectioned, stained with 0.5% toluidine blue and examined under a light microscope (BX51, Olympus, Tokyo, Japan). Ultrathin sections were stained with 5% uranylacetate and Reynold’s lead citrate, then observed and photographed under the transmission electron microscope (JEM-1200EXII, JEOL Co., Tokyo, Japan).

### 4.9. Irradiation of Cells and Colony Formation Analysis

CT26 cells were seeded in culture dishes at a density of 150 cells per dish in RPMI-1640 medium containing 10% FCS at 37 °C in a humidified 5% CO_2_ incubator. Cells were treated with various concentrations of DBT-PD (0, 0.33, 1.0 and 3.3 mg/mL) for 24 h. DBT-PD was then washed out, and the cells were irradiated with various doses (0, 0.5, 1, 2, 3, and 4 Gray (Gy)) using a 6 MeV electron beam generated by a linear accelerator (Clinac^®^ 1800, Varian Associates, Inc., Palo Alto, CA, USA) with a dose rate of 2.4 Gy/min in a single fraction. Full electron equilibrium was ensured for each fraction by a parallel plate PR-60C ionization chamber (CAPINTEL, Inc., Ramsey, NY, USA). Radiation doses were selected based on our preliminary radiation survival curves using CT26 cells in the absence of DBT-PB. This data allowed us to identify a dose range that killed 0 to over 63% of cells. After 10–14 days, cells were stained with 3% crystal violet and the number of colonies (≥50 cells) was counted. The surviving fraction was calculated as cells inoculated × plating efficiency. To assess the effect of DBT-PD plus concurrent chemoradiotherapy, an irradiation dose of 2 Gy was selected to match the daily fraction size commonly used in clinical practice. CT26 cells were cultured under 0.25 μM 5-FU and 1.0 or 3.3 mg/mL DBT-PD for 24 h then exposed to 2 Gy irradiation. Colony formation and surviving fraction were calculated as described above.

### 4.10. Statistical Analysis

All experiments were performed and repeated at least three times. Data were expressed as mean ± standard deviation from three independent experiments and were analyzed via analysis of variance with post-hoc comparison between control and treated groups. Statistically significant values were indicated by *p* < 0.05. Combination index values were calculated using the CompuSyn Version 5.20.2010 program according to Chou et al. [25,26].

## 5. Conclusions

DBT-PD induced autophagy-associated cell death in colorectal cancer CT26 cells. It may have therapeutic potential as a chemotherapy and RT sensitizer in colorectal cancer treatment, and an important addition to the arsenal of integrative medicine practitioners.

## Figures and Tables

**Table 1:** Index component analysis of DBT, DBT-PE and DBT-PD.

Index Component	DBT	DBT-PE	DBT-PD
Ferulic acid (µg/g)	0.294	0.433	1.404
Astragaloside IV (µg/g)	48.462	11.367	312.626

**Table 2:** Osmolality measurement of culture medium with different additives.

Control	1/2000 Ethanol	DBT-PD 3.3 mg/mL	DBT-PD 10 mg/mL	DBT-PD 15 mg/mL	DBT-PD 30 mg/mL
291.0 ± 5.2	375.3 ± 34.1	370.7 ± 11.0	360.0 ± 21.7	387.3 ± 14.4	507.7 ± 18.8

Unit: mOsm/kg; Osmolality of culture solutions (with or without 0–30 mg/mL DBT-PD) was measured on an osmometer, which works based on the method of depression of freezing point. The osmolality of culture medium without additives was 291.0 ± 5.2 mOsm/kg. After mixing with ethanol or different concentrations of DBT-PD, osmolality increased to around 370 mOsm/kg for DBT-PD concentrations under 15 mg/mL.

**Table 3:** Caspase activity measurement in CT26 cells treated with DBT-PD.

Caspase	Control	DBT-PD 10 mg/mL
1	28.00 ± 2.76	23.07 ± 2.66
2	32.05 ± 6.72	31.20 ± 4.17
3/7	30.49 ± 6.91	29.87 ± 7.85
4	33.18 ± 1.63	33.26 ± 3.57
5	21.39 ± 4.02	20.56 ± 2.92
6	27.81 ± 1.92	27.81 ± 2.14
8	23.72 ± 0.75	20.60 ± 0.50
9	22.09 ± 1.84	22.94 ± 5.54
10	25.38 ± 8.83	24.53 ± 4.71

Units: relative fluorescence units. Caspase activities were measured after CT26 cells were administered vehicle and DBT-PD. Cell lysates were incubated with substrates conjugated with AFC. Different caspase activities in CT26 cells were not altered with or without DBT-PD treatment.

**Table 4:** Percent growth inhibition and combination index (CI) of CT26 cells treated with DBT-PD and different concentrations of 5-FU.

Treatment Time	DBT-PD (mg/mL)	5-FU (μM)			
		0.0	0.1	0.5	1.0
24 h	0.0	-	6.7% ± 1.6%	58.6% ± 11.2%	76.3% ± 9.2%
	1.0	30.5% ± 1.0%	29.7% ± 3.0% (1.45)	67.2% ± 1.6% (0.82)	77.7% ± 8.3% (1.11)
	3.3	48.8% ± 14.8%	62.4% ± 7.2% (0.61)	84.0% ± 6.0% (0.49)	80.8% ± 6.5% (1.06)
48 h	0.0		10.9% ± 9.6%	76.0% ± 8.4%	91.7% ± 2.9%
	1.0	34.1% ± 6.1%	51.5% ± 5.7% (0.87)	83.1% ± 5.1% (0.93)	94.4% ± 2.5% (0.94)
	3.3	68.9% ± 3.4%	74.2% ± 4.3% (1.01)	95.9% ± 0.9% (0.53)	96.2% ± 2.9% (0.84)
72 h	0.0		16.4% ± 4.9%	83.5% ± 4.4%	95.6% ± 1.9%
	1.0	34.1% ± 8.7%	37.7% ± 6.9% (1.46)	88.4% ± 3.9% (0.93)	96.8% ± 2.5% (0.86)
	3.3	65.8% ± 12.4%	72.0% ±7.0% (1.10)	97.7% ± 1.5% (0.42)	98.4% ± 0.3% (0.66)

Concurrent treatment of CT26 cells with different concentrations of 5-FU and DBT-PD inhibited growth more strongly than 5-FU alone. Strikingly, 5-FU concentrations significantly lower than the normal dosing regimen (0.1 and 0.5 μM) were more effective in the presence of 3.3 mg/mL DBT-PD by 55.7% and 25.4% after 24 h. The CIs for concurrent treatment of 5-FU and DBT-PD were shown below the growth inhibition percentage, whereas CI < 1 indicated synergistic effect; CI > 1 indicated antagonism.

**Table 5:** Percent growth inhibition and combination index (CI) of HT-29 cells treated with DBT-PD and different concentrations of 5-FU.

Treatment Time	DBT-PD (mg/mL)	5-FU (μM)			
		0.0	0.1	0.5	1.0
72 h	0.0		27.4% ± 10.0%	38.1% ± 14.8%	66.4% ± 4.3%
	1.0	22.2% ± 11.3%	42.4% ± 5.0% (0.65)	59.7% ± 9.6% (0.72)	66.5% ± 3.5% (0.77)
	3.3	46.4% ± 7.6%	59.1% ± 10.2% (0.75)	34.5% ± 4.6% (4.07)	73.1% ± 5.1% (0.73)

Concurrent treatment of HT-29 cells with different concentrations of 5-FU and DBT-PD more strongly inhibited growth than 5-FU alone did after 72 h. The 5-FU concentrations (significantly lower than the normal dosing regimen (0.1 and 0.5 μM)) were more effective in the presence of 1.0 and 3.3 mg/mL DBT-PD after 72 h. The CIs of concurrent treatment of 5-FU and DBT-PD were below the growth inhibition percentage, whereas CI < 1 indicated synergistic effect; CI > 1 indicated antagonism.

## Supplementary Materials

Supplementary materials can be accessed at: http://www.mdpi.com/1420-3049/21/12/1677/s1.
