# Supplementary material for: The Traditional Chinese Medicine DangguiBuxue Tang Sensitizes Colorectal Cancer Cells to Chemoradiotherapy
Source: Molecules. 2016 Dec 6;21(12):1677. doi: 10.3390/molecules21121677 (PMC6273051; doi:10.3390/molecules21121677)
Supplement: Supplementary file 1 [file molecules-21-01677-s001.pdf]

# Supplementary Materials: Traditional Chinese Medicine DangguiBuxue Tang Sensitizes Colorectal Cancer Cells to Chemoradiotherapy

**Shun-Ting Chen, Tzung-Yan Lee, Tung-Hu Tsai, Yin-Cheng Lin, Chin-Ping Lin, Hui-Ru Shieh, Ming-Ling Hsu, Chih-Wen Chi, Ming-Cheng Lee, Hen-Hong Chang and Yu-Jen Chen**

## Material and Method

### *Animal Study*

A subcutaneous xenograft animal model was used to examine the tumor growth inhibition and for the radiosensitization assay. Male BALB/c mice, aged 6 to 8 weeks, were obtained from the National Laboratory Animal Center (Taipei, Taiwan) and housed in a rodent facility under a 12-h light-dark cycle in pathogen-free conditions. All experiments were performed in accordance with the regulations of the NIH Guide for the Care and Use of Laboratory Animals. Four groups each of three mice were subcutaneously injected with CT26 cells ($2.5 \times 10^5$ cells) into the right gluteal region. Treatment was started on day 7, so that the tumors were approximately 0.5 cm in diameter. The mice were subgrouped into vehicle, DBT-PD, irradiation (RT), and RT plus DBT-PD. Local radiotherapy was delivered with 3 Gy in single fractions on the first day of treatment. Mice were orally fed 0.39 g/kg DBT-PD for four consecutive weeks. The tumor size of each mouse was measured with a caliper and was calculated using the following formula:

$$a \times b^2/2 \quad (1)$$

where $a$ is the largest and $b$ is the smallest diameter. The tumor size was estimated every 2–3 days

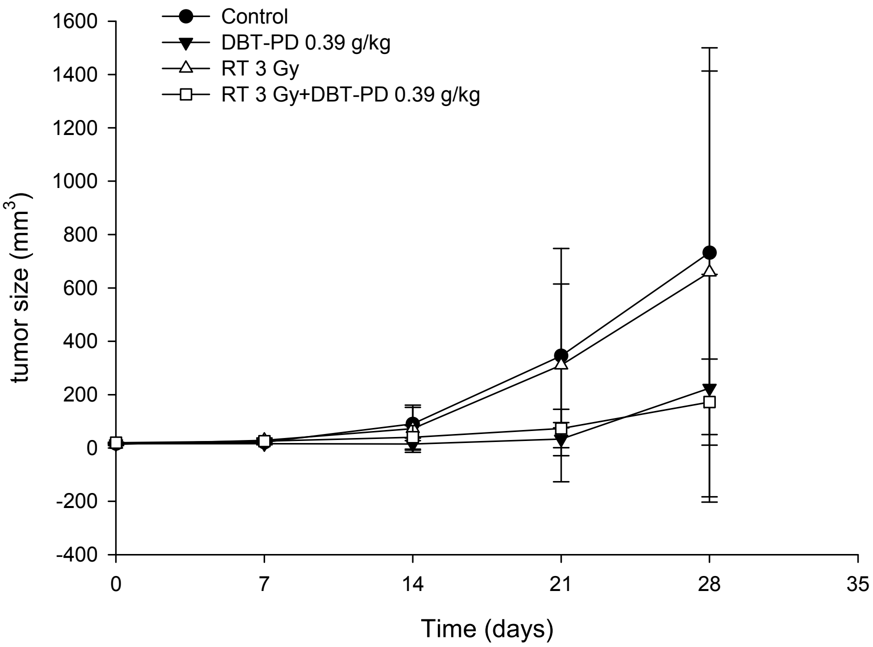


**Figure S1.** In vivo effect of DBT-PD and radiation (RT) on tumor growth. CT26-bearing BALB/c mice were treated with vehicle (control), orally fed 0.39 g/kg DBT-PD via tubes with for 4 weeks, 3 Gy in single fraction in first week, or DBT-PD plus RT. Data from three mice in each group are presented as mean ± standard deviation.
